# Modulation of *Cerrena unicolor* laccase and manganese peroxidase production

**DOI:** 10.1186/2193-1801-3-463

**Published:** 2014-08-26

**Authors:** Eva Kachlishvili, Eka Metreveli, Vladimir Elisashvili

**Affiliations:** Agricultural University of Georgia, 240 David Agmashenebeli alley, 0159 Tbilisi, Georgia

**Keywords:** *Cerrena unicolor*, Carbon source, Inducers, Lignocellulosic materials, Laccase, Manganese peroxidase, Submerged fermentation

## Abstract

Among seven carbon sources tested, glycerol and glucose favored the *Cerrena unicolor* laccase production (18.8-20.3 U/mL); in addition, glycerol ensured the highest manganese peroxidase (MnP) activity (2 U/mL). Substitution of glycerol with the ethanol production residue (EPR) gave the highest laccase (90.1 U/mL) activity, while the walnut pericarp provided the highest MnP activity (7.4 U/mL). Supplementation of medium with 1 mM copper and 1 mM xylidine at appropriate time caused significant additive effect on laccase expression (333.2 U/mL) in shake-flask experiments. Overproduction of laccase activity (507 U/mL) and secretion of MnP activity was obtained when *C. unicolor* was cultivated in stirred-tank fermenter. *C. unicolor* showed several distinctive and attractive technological features: it is capable to synthesize high levels of oxidases under high carbon and high nitrogen conditions and it secretes high laccase activity during trophophase.

## Introduction

White-rot basidiomycetes (WRB) are known to be efficient lignocellulose degraders and they are recognized for their unique capability to degrade lignin secreting lignin peroxidase (EC 1.11.1.14), manganese-dependent peroxidase (EC 1.11.1.13), and laccase (EC 1.10.3.2), which function together with hydrogen peroxide-producing oxidases (Aro et al. 2005). Besides their fundamental importance for efficient bioconversion of plant residues in nature, lignin-modifying enzymes (LME) may have a large variety of biotechnological and environmental applications requiring huge amounts of these biocatalysts at a low cost (Madhavi and Lele 2009). These demands have intensified search for new potent fungi overproducing LME with industrially relevant properties. However, among the hundreds tested strains belonging to different taxonomic and ecological groups only few WRB showed high potentials for the production of the oxidative enzymes (Galhaup et al. 2002; Revankar and Lele 2006; Songulashvili et al. 2012). Various approaches and strategies, such as supplementation of nutrient media with food industry wastes/by-products, favorable carbon and nitrogen sources at an appropriate concentration, addition of effective inducers have been explored to accelerate the LME synthesis and to increase their yield (Galhaup et al. 2002; Kapich et al. 2004; Michniewicz et al. 2006; Revankar and Lele 2006; Elisashvili et al. 2011).

There are few reports on the production of LME by the *Cerrena unicolor* strains (Janusz et al. 2007; Lisova et al. 2010; Songulashvili et al. 2012; Hibi et al. 2012; Rola et al. 2013). However, the knowledge related to the physiological regulation of *C. unicolor* LME synthesis is limited to develop economical ways of industrial scale enzyme production. Especially scarce are data on the MnP synthesis by this fungus (Elisashvili et al. 2011; Hibi et al. 2012). Hence, the main objective of the present work was to elucidate the features and modulation of *C. unicolor* CBS 117347 LME activity in response to different lignocellulosic materials, carbon sources, and inducers. Taking into account the potential applications of laccase in various biotechnologies, the enzyme production was scaled up in the laboratory fermenter employing the developed medium composition and creating cultivation conditions favorable for the target enzyme secretion.

## Materials and methods

### Organism and inoculum preparation

*C. unicolor* CBS 117347 inocula were prepared by growing the fungal mycelium from agar slant on a rotary shaker at 27°C and 150 rpm in 250-mL flasks containing 100 mL of the medium (per liter): 10 g glucose, 2 g NH_4_NO_3_, 1 g KH_2_PO_4_, 0.5 g MgSO_4_ · 7H_2_O, 2 g yeast extract. After 7 days of cultivation the fungal biomass was homogenized in a Waring laboratory blender.

### Cultivation conditions in shake-flasks experiments

The submerged cultivation of fungus was conducted in Innova 44 shaker (New Brunswick Scientific, USA) at 27°C and 150 rpm. The homogenized mycelium (3 mL) was used to inoculate the 250-mL flasks containing 50 mL of the basal medium (per liter): 1 g KH_2_PO_4_, 0.5 g MgSO_4_ · 7H_2_O, 0.07 g CuSO_4_ · 5H_2_O, 2 g NH_4_NO_3_, 3 g yeast extract. Crystalline cellulose, xylose, glucose, sucrose, glycerol, mannitol, and sodium gluconate in concentration of 15 g/L were used as carbon sources and mandarin peels, ethanol production residue (EPR) from the wheat grains, wheat bran, banana peels, leaves of beech, and walnut pericarp in an amount of 40 g/L were used as growth substrates. All plant residues were dried at 50°C and milled to powder.

To establish the role of water-soluble (extractable) compounds and insoluble residue of EPR as well as localization/origin of compounds promoting the target LME expression the following treatment was performed. 40 g EPR were boiled in 1 L of above-mentioned basal medium during 5 min on magnetic stirrer. Then the residue was separated from the liquid extract by filtration, washed with the same volume of basal medium and separated by centrifugation followed by drying at 70°C. The liquid fractions (extracts) were combined and used directly as a nutrient medium for the fungus cultivation while the extracted and dried EPR was added to the new basal medium in concentration of 40 g/L.

To evaluate the effect of copper and to study the synergistic effect of copper and 2,5-xylidine, both compounds were added in concentration of 1 mM to the 5% EPR-containing basal medium without copper (control) before inoculation or on the third day of cultivation. The initial pH of all media was adjusted to 6.0 prior to sterilization. The fungus submerged cultivations were carried out during 14 days. At predetermined time intervals, 1-mL samples were taken and the solids were separated by centrifugation (Eppendorf 5417R, Germany) at 10,000 *g* for 5 min at 4°C. The supernatants were analyzed for pH, reducing sugars and enzyme activities. All experiments were performed twice using three replicates each time. Data presented correspond to the mean values with the standard deviations being less than 20%.

### Cultivation in fermenter

To scale up the *C. unicolor* laccase production the fungus cultivation was performed in 12 L BioFlo Fermenter 2000 (New Brunswick Scientific, USA) with three Rushton impellers. The fermenter was filled with 8 L of the optimized medium (g/L): EPR - 50, peptone – 5; KH_2_PO_4_ – 1, MgSO_4_ · 7H_2_O – 0.5, yeast extract – 5, polypropylene glycol 2000 – 8 mL, pH – 5.5. After two days of fermentation solutions of xylidine and Cu^2+^ were added to the fermenter to the final concentrations of 1 mM. The fermenter equipped with pH, temperature and pO_2_ probes was sterilized (121°C, 40 min) and inoculated with homogenized mycelium (10% of total volume to accelerate fermentation). Fermentation was carried out without baffles at 27°C, impeller speed 300 rpm, airflow rate 0.5 v/v/min, and pH 5.5. During fermentation process samples were collected daily and analyzed for enzyme activity and purity using a light microscope.

### Analytical methods

The total nitrogen was determined according to the Kjeldahl method with Nessler reagent after pre-boiling of samples in 0.5% solutions of trichloroacetic acid for 15 min to remove non-protein content. True protein was calculated as the total nitrogen multiplied by 4.38.

Laccase activity was determined spectrophotometrically (Camspec M501, UK) at 420 nm as the rate of 0.25 mM ABTS (2,2′-azino-bis-[3-ethyltiazoline-6-sulfonate]) oxidation in 50 mM Na-acetate buffer (pH 3.8) at room temperature (Bourbonnais and Paice 1990). MnP activity was measured at 270 nm by following the formation of a Mn^3+^-malonate-complex (Wariishi et al. 1992). One unit of laccase or MnP activity was defined as the amount of enzyme that oxidized 1 μmoL of substrate per minute.

## Results

### Effect of carbon source

Table 1 shows that all tested soluble compounds supported comparatively good growth of *C. unicolor* CBS 117347 resulting in 4.2-6.8 g biomass/L. The maximum biomass yield was observed in media containing mannitol followed by glycerol and glucose. Biomass yield was significantly reduced in the sodium gluconate-based medium, but it was 6-fold higher than that in the basal medium containing 0.3% yeast extract as the only carbon source.

**Table 1:** **Effect of carbon sources on the** ***C. unicolor*** **laccase and MnP activity**

Carbon source	Biomass (mg/mL)	Laccase		MnP	
		(U/mL)	(U/mg)	(U/mL)	(U/mg)
Control	0.7 ± 0.10	0.7 ± 0.1^7b^	1.0	0.2 ± 0.02^7^	0.29
Avicel	3.0 ± 0.23^a^	6.8 ± 1.3^14^	2.3	0.4 ± 0.05^4^	0.13
Xylose	5.2 ± 0.33	17.0 ± 2.6^10^	3.3	1.1 ± 0.08^10^	0.21
Glucose	6.0 ± 0.31	20.3 ± 3.2^10^	3.4	1.2 ± 0.16^7^	0.19
Sucrose	4.7 ± 0.40	15.8 ± 1.9^10^	3.4	0.3 ± 0.04^7^	0.06
Na gluconate	4.2 ± 0.27	6.5 ± 1.0^7^	1.5	0.6 ± 0.09^7^	0.15
Mannitol	6.8 ± 0.41	10.6 ± 1.3^14^	1.6	1.1 ± 0.09^10^	0.16
Glycerol	6.5 ± 0.45	18.8 ± 2.7^10^	2.9	2.0 ± 0.34^7^	0.31

Carbon sources concentration was 15 g/l.

Samples were taken after 4, 7, 10, and 14 days of submerged cultivation.

Values presented are the means ± SD of two experiments with three replicates.

^a^Calculated from protein content.

^b^The numbers indicate the days of the peak activity.

Maximum laccase activity was revealed in the glucose containing medium while the highest MnP activity (2 U/mL) was achieved in medium with glycerol. Xylose, sucrose, and glycerol also favored laccase production. Substitution of these carbon sources with sodium gluconate or crystalline cellulose caused decrease of *C. unicolor* laccase activity from 15.8-20.3 U/mL to 6.5-6.8 U/mL. Sucrose appeared to be poor carbon source for MnP activity expression.

### Effect of lignocellulosic substrate

In this study, the potential of *C. unicolor* to secrete LME was evaluated in submerged cultivation of fungus in glycerol- and lignocellulose-containing media. All materials supported equally good fungal growth in form of small pellets. However, the values for individual oxidases differed significantly depending on growth substrates used (Table 2). Among lignocellulosic materials tested, EPR, wheat bran, mandarin and banana peels appeared to be the most preferred substrates for laccase production by this fungus yielding 77.9-90.1 U/mL, approximately 3-fold higher enzyme activity as compared with that in walnut pericarp and beech leaves-containing media. By contrast, the walnut pericarp provided the highest MnP activity of *C. unicolor* (7.4 U/mL after 10 days of submerged fermentation).

**Table 2:** ***C. unicolor*** **laccase and MnP activity in submerged fermentation of lignocellulosic substrates**

Substrates	Laccase (U/mL)	MnP (U/mL)
Glycerol	20.5 ± 3.4^10a^	2.2 ± 0.46^10^
Banana peels	77.9 ± 14.0^7^	2.6 ± 0.44^7^
Beech leaves	23.1 ± 3.8^7^	0.8 ± 0.12^7^
EPR	90.1 ± 13.3^10^	1.6 ± 0.25^7^
Mandarin peels	87.7 ± 14.3^7^	2.1 ± 0.35^7^
Walnut pericarp	35.0 ± 5.8^7^	7.4 ± 1.38^10^
Wheat bran	89.6 ± 17.0^10^	1.0 ± 0.13^7^

Lignocellulosic substrates concentration was 40 g/l.

Samples were taken after 4, 7, 10, and 14 days of submerged fermentation.

Values presented are the means ± SD of two experiments with three replicates.

^a^The numbers indicate the days of the peak activity.

**Figure 1:**
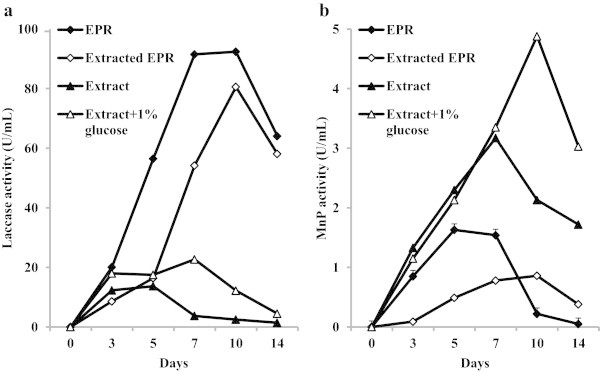
**Effect of solid and water-soluble fractions of EPR on the** ***C. unicolor*** **laccase (a) and MnP (b) activities.**

It was interesting to establish the origin of EPR compounds promoting the LME expression. For this reason, the fungus was cultivated in the basal media containing 1) EPR; 2) the water extracted EPR; 3) water extract from EPR. Significant laccase activity was observed in all tested media already after 3 days of fungus cultivation (Figure 1). However, the maximum enzyme activity in EPR-containing medium was reached after 7 days of *C. unicolor* cultivation while in medium with the washed substrate it was achieved three days later in addition to slower enzyme secretion. The extractives-containing medium favored rapid accumulation of laccase during first 3 days and supplementation of this medium with additional carbon source further increased the enzyme yield. Measurement of *C. unicolor* MnP activity showed that the substitution of EPR with washed substrate delayed enzyme secretion during initial three days and two-fold decreased the enzyme yield. By contrast, the water extract accelerated the MnP production and significantly increased the enzyme yield.

### Enhancement of laccase production by copper and xylidine

A poor medium and consequently low biomass yield are impractical for the production of high yield of LME. According to our preliminary experiments, conditions promoting laccase expression by *C. unicolor* CBS 117347 appeared to be different to those previously reported for many other fungi. In particular, laccase production by this fungus was enhanced in presence of high concentrations of organic nitrogen source, copper, and xylidine. Therefore, based on these data, the subsequent cultivations of fungus were performed in nutritionally richer medium. In particular, peptone in concentration of 5 g/L was added to the nutrient medium instead ammonium tartrate while the yeast extract and EPR concentrations were increased from 3 g/L and 40 g/L to 5 g/L and to 50 g/L, respectively. Copper and xylidine were supplemented to the medium separately and in combination on the day of inoculation and after 3 days of fermentation when the culture was in the exponential phase of growth.

**Figure 2:**
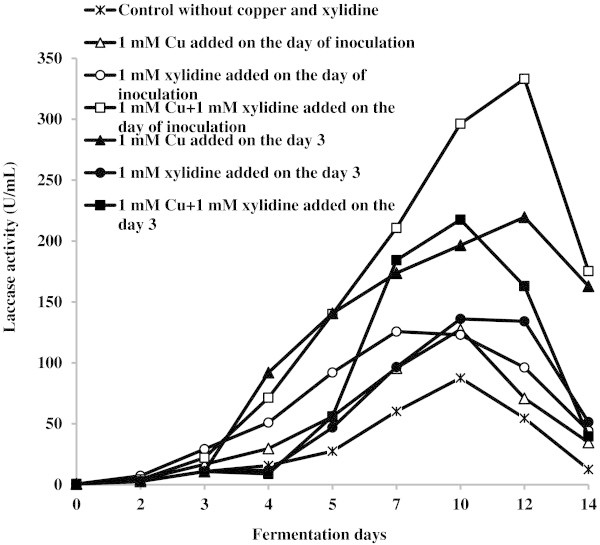
**Effect of copper and xylidine on the** ***C. unicolor*** **laccase activity in submerged fermentation of EPR.**

As it can be observed in Figure 2, the control medium without copper and xylidine provided an accumulation of 87.6 U laccase activity/mL after 10 days of cultivation. Supplementing the culture medium with copper or xylidine separately significantly accelerated enzyme secretion from the second day of fermentation and 1.8-fold improved laccase yield (to 127.4 U/mL after 10 days and to 125.7 U/mL after 7 days, respectively). Substantial increase of laccase activity (to 333.2 U/mL after 12 days) and synergistic effect was observed when copper and xylidine have been supplemented to the medium simultaneously before inoculation. Addition of the selected inducers to actively growing culture resulted in further increase of laccase yield, especially in the presence of Cu^2+^. By contrast, no stimulation of *C. unicolor* MnP activity was observed during entire period of fungus cultivation in media supplemented with copper or xylidine. Enzyme activity (0.15-0.42 U/mL) of fungus grown in presence of inducers appeared to be lower as compared with that in control medium (0.54 U/mL) indicating that expression of this enzyme is independent on the tested compounds. Moreover, low MnP activity can be explained by the fact that the enzyme synthesis is suppressed in the presence of high concentrations of nutrient carbon and nitrogen.

### Laccase and MnP production in fermenter

To scale up laccase production by *C. unicolor* CBS 117347 the fungus cultivation was carried out in a fermenter filled with an optimized medium, taking into account the results obtained in agitated flask cultures. A stirred-tank bioreactor was employed to efficiently produce the target enzyme since it provides better mixing of medium and the fungus growth in form of pellets of desired size. Several preliminary fermentations have been performed to establish the most appropriate agitation and aeration rates as well as the time of xylidine and copper addition to the growing culture. The highest laccase activity was achieved when the agitation rate was 300 rpm while the aeration rate was 0.5 L/L/min during entire fermentation. Application of these conditions prevented mycelium sedimentation and provided sufficient dispersion of air. Moreover, the medium pH was maintained automatically at value 5.5 during entire fermentation process. The maintenance of medium pH on this level favored hydrolyses of EPR polysaccharides to steadily supply the growing fungus with carbon and energy source. In addition, this value of pH is the most appropriate for the stability of already synthesized enzyme.

**Figure 3:**
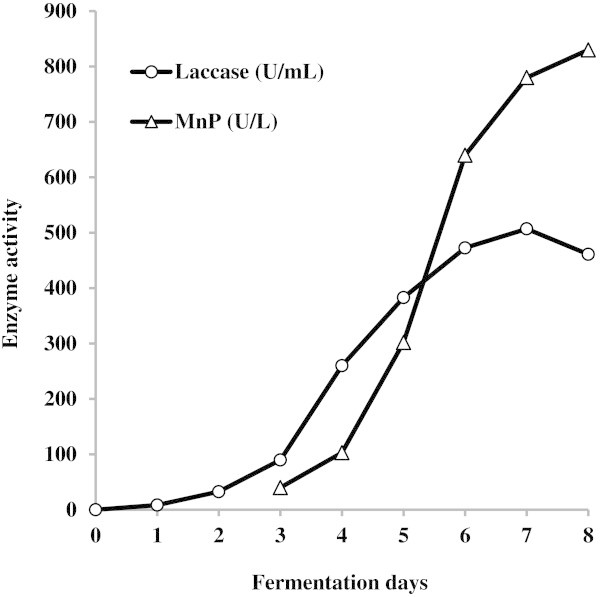
**Time course of a typical** ***C. unicolor*** **laccase and MnP production in a stirred tank fermenter.**

Significant laccase activity (8.4 U/mL) was detected already after 24 h fermentation of EPR (Figure 3). Subsequently, after addition of 1 mM copper sulphate and 1 mM xylidine on day 2, it gradually increased with highest productivity during an exponential phase of growth and peaked (507 U/mL) after 7 days of fungus cultivation. It is interesting that the low MnP activity (0.04 U/mL) was detected after 3 days of *C. unicolor* cultivation, but the enzyme activity continued to increase till the end of fermentation (0.83 U/mL).

## Discussion

In this work, we described several distinctive and attractive technological features of *C. unicolor* CBS 117347. Firstly, this fungus is an outstanding laccase producer. EPR, wheat bran, and mandarin peels provided the best laccase production yielding as high as 87.5-90.1 U/mL. Moreover, the data received indicate that *C. unicolor* is a promising producer of MnP and walnut pericarp used for the first time as a growth substrate gave very high yield of MnP manifold exceeding that in other media and other studies (Kapich et al. 2004; Songulashvili et al. 2007; Elisashvili and Kachlishvili 2009). Secondly, *C. unicolor* is capable of synthesizing LME in both synthetic and lignocellulose-containing media. Considerable levels of laccase and MnP have been revealed in the presence of all tested carbon sources, including mono-, di-, and polysaccharides, sugar alcohols and organic acid. In this respect, the ligninolytic system of fungus differed from that of *Phanerochaete chrysosporium* (Kapich et al. 2004), *Ganoderma lucidum* (Songulashvili et al. 2007), *G. applanatum*, *G. tsugae*, and *Pleurotus tuber-regium* (Elisashvili and Kachlishvili 2009) which were unable to produce appreciable laccase or peroxidases activities in synthetic media. Moreover, Michniewicz et al. (2006) showed that *C. unicolor* C-137 did not secrete peroxidase activity neither in synthetic glucose containing Kirk medium nor in complex tomato juice containing medium even in the presence of manganese, known to stimulate the production of manganese peroxidase in some basidiomycetes. However, our finding is in good agreement with observations of other researchers. For example, Hibi et al. (2012) showed that *Cerrena* sp. was capable of secreting laccase and three peroxidases in submerged cultivation in a glucose-containing medium. In addition, *C. unicolor* C-139 accumulated 18 nkat/mL (Janusz et al. 2007) and 28 nkat/mL (Rola et al. 2013) laccase activities in the synthetic media with glucose and maltose as a carbon source, respectively. Thirdly, *C. unicolor* CBS 117347 is capable to synthesize high levels of LME under high carbon and high nitrogen conditions and it secretes high laccase activity during trophophase.

*C. unicolor* CBS 117347 enzyme activity is highly influenced by different carbon sources. Among carbon sources tested, maximum laccase activity was observed in glucose containing medium while the highest MnP activity was achieved in medium with glycerol. Substitution of glucose by sodium gluconate decreased *C. unicolor* laccase activity from 20.3 U/mL to 6.5 U/mL (Table 1). It is worth noting that the fungal biomass yield in glucose-containing medium (6 mg/mL) was only 1.4-fold higher as compared with that in gluconate-based medium (4.2 mg/mL) whereas the laccase specific activity in former medium (3.4 U/mg biomass) appeared to be more than 2-fold higher than that in the last medium (1.5 U/mg biomass). Moreover, our calculations showed that the MnP producing ability of fungus varied from 0.06 U/mg in medium with sucrose to 0.31 U/mg in medium supplemented with glycerol. Furthermore, enzyme accumulation profiles differed significantly in fungus cultivation in tested media. *C. unicolor* growth in xylose-based medium accompanied with intensive secretion of laccase and on the 4th day of inoculation this enzyme activity attained 9.9 U/mL while in other media it ranged from 1.1 to 2.3 U/mL. On the contrary, in crystalline cellulose and mannitol-containing media laccase activity slowly accumulated during 10 days of growth and peaked on day 14. Finally, in medium supplemented with sucrose laccase activity increased gradually over 10 days of growth, and then decreased until the end of the cultivation. It means that the enzyme accumulation profile should be always monitored to correctly evaluate the producer biosynthetic potential.

Analysis of data received shows that *C. unicolor* CBS 117347 is capable to constitutively produce laccase and MnP in the control medium with the specific activity of 1 U/mg and 0.29 U/mg, respectively. Nevertheless, it is not excluded that in this case, the enzyme production may be attributed to the availability of a pool of specific amino acids present in yeast extract. Supplementation of control medium with carbon source provided significant increase of volumetric activity of both enzymes. However, the comparison of the MnP specific activities evidences that the higher enzyme activity was due to a higher biomass production in the media supplemented with carbon sources. Moreover, it seems that the MnP secretion was rather inhibited in their presence since the specific activity of MnP (0.06-0.21 U/mg) in all media, with the exclusion of glycerol, appeared to be much lower than that in control. By contrast, the laccase specific activity increased 1.5-3.4-fold in all tested media. These findings indicate that carbon source somehow influence enzyme expression although a fundamental question arises why do fungi produce high levels of laccase and MnP in absence of the natural substrate and why enzyme activity is modulated in dependence on nature of carbohydrate? Obviously, the consumption rate of individual carbohydrates plays an essential role. Galhaup et al. (2002) concluded that an important factor for efficient laccase production by *T. pubescens* is the concentration of glucose since enhanced production of laccase only occurred when the glucose concentration had dropped below a low, critical value. Analogically, the maximal laccase activity of *C. unicolor* C-139 was observed at the end of exponential growth phase when maltose concentration dropped down to 10% of initial amount (Rola et al. 2013).

In this study, based on earlier work (Elisashvili et al. 2006; 2011; Kachlishvili et al. 2012), mandarin peels, EPR, wheat bran, and banana peels containing high concentrations of carbohydrates and aromatic compounds have been selected to assess the capability of *C. unicolor* CBS 117347 to produce LME. In addition, leaves of beech tree and walnut pericarp also were tested as new growth substrates and source of inducers for the target enzymes synthesis. The results herein described demonstrate a clear regulatory role and significant stimulation of laccase and MnP activity by several lignocellulosic substrates (Table 2). Substitution of glucose with EPR increased the specific laccase activity from 3.4 to 19 U/mg biomass. The water-extracted EPR provided even higher specific activity of laccase (29 U/mg). However, the laccase synthesis in this medium was significantly delayed and the comparatively high maximum enzyme activity was attained only 5 days later (Figure 1). Hence, some compound promoting enzyme secretion appeared during EPR submerged fermentation and accelerated laccase synthesis. At the same time, the extract-based medium obviously contained small amount of laccase synthesis inducer that favored rapid accumulation of enzyme during first 3 days. Nevertheless, this medium was poor with carbon source to produce high levels of biomass and laccase as the specific enzyme activity was only 8 U/mg. Supplementation of this medium with 1% glucose further increased the laccase yield, but this increase was due to the higher biomass accumulation.

The measurement of *C. unicolor* MnP activity showed that the substitution of EPR with the washed substrate delayed enzyme secretion and two-fold decreased the enzyme yield. By contrast, the extractive substances derived from EPR significantly accelerated the MnP production already during first days of fungus cultivation and significantly increased the enzyme yield. It is worth noting that the specific MnP activity increased from 0.34 U/mg in EPR-containing medium to 1.76 U/mg in the extractives-based medium. Thus, the data obtained strongly indicate that some water-soluble aromatic/phenolic compounds present in the EPR or deriving from this material during fermentation may function as inducers for the LME production by *C. unicolor*. This assumption as well as a bioavailability of such compounds should be explored in further experiments.

Genes encoding LME of the white-rot fungi have been found to be differentially regulated in response to a wide variety of environmental signals (Janusz et al. 2013). Among them, copper and aromatic compounds are considered as the most potent inducers of enzyme synthesis (Revankar and Lele 2006; Elisashvili and Kachlishvili 2009; Lisova et al. 2010; Janusz et al. 2013). In this study, supplementing the culture medium with 1 mM CuSO_4_ or 1 mM xylidine significantly accelerated laccase secretion and increased enzyme yield (Figure 2). Moreover, additive effect on the laccase expression was observed when copper and xylidine were supplemented to the medium simultaneously. It is worth noting that addition of xylidine or this compound in combination with copper to actively growing culture completely repressed laccase production during at least 24 h. Subsequently, the enzyme secretion resumed and during the subsequent 24 h of fermentation it enhanced 4-fold and 5-fold, respectively. It is possible that the enhanced synthesis of laccase is a response of *C. unicolor* to chemical stress in presence of high concentrations of two toxic compounds. The stimulating effect of copper on the laccase production by *C. unicolor* strains was described by other authors (Michniewicz et al. 2006; Janusz et al. 2007; Lisova et al. 2010). However, the addition of only 50 μM copper stimulated *C. unicolor* strain 137 laccase expression up to 100-fold and resulted to a maximum enzyme level of 4 U/mL; higher concentrations of Cu^2+^ (0.2 mM) did not further stimulate laccase activity (Michniewicz et al. 2006). Moreover, all the tested aromatic compounds were ineffective as inducers of laccase synthesis by *C. unicolor* VKM F-3196 while copper in concentration of 0.1 mM provided 15 U laccase activity/mL on the 8th day of fungus cultivation (Lisova et al. 2010).

Finally, using data obtained and different approaches, overproduction of laccase activity (507 U/mL) and secretion of MnP activity have been achieved in a relatively short time when *C. unicolor* CBS 117347 was cultivated in stirred-tank fermenter. Previously, comparatively low laccase activity was revealed in synthetic medium in shaken flask cultures (6 U/mL) and in fermenter (3.9 U/mL) (Janusz et al. 2007), as well as in a complex tomato juice medium (19 U/mL) (Michniewicz et al. 2006); however, the highest laccase activity (450 U/mL) among *Cerrena* species tested so far was achieved in submerged fermentation of wheat bran by *C. unicolor* strain C-139 (Songulashvili et al. 2012). Based on the optimized induction conditions developed here laccase production by *C. unicolor* CBS 117347 could be further scaled up for different biotechnological applications. Moreover, this fungus is a suitable system to understand the LME expression in other basidiomycetous fungi.
